# Integrative multi-omics analysis of the microbiome and metabolome in bronchoalveolar lavage fluid from patients with early-stage lung cancer

**DOI:** 10.3389/fcimb.2025.1513270

**Published:** 2025-04-28

**Authors:** Jiajun Xie, Nengyang Zhu, Weiguo Xu

**Affiliations:** Department of Respiratory and Critical Care Medicine, Mianyang Central Hospital, School of Medicine, University of Electronic Science and Technology of China, Mianyang, China

**Keywords:** bronchoalveolar lavage fluid (BALF), lung cancer, LC-MS, 16S rDNA, SPN

## Abstract

Lung cancer is a significant health concern that poses a considerable threat to human health and quality of life. In order to enhance the prognosis of patients with lung cancer, we conducted a combined analysis of 16S rDNA gene sequencing of alveolar lavage fluid and LC-MS metabolomics research, with the objective of identifying biomarkers in patients with early-stage lung cancer presenting as SPN. A comparison of the benign nodule group and the early-stage lung cancer patients revealed that the phylum-level Bacteroidetes and the genus-level Chryseobacterium and Delftia were more abundant in the latter group. Additionally, the Fusobacteriales might serve as a predictive marker for the diagnosis of early-stage lung cancer. In the context of metabolomics, the early-stage lung cancer was found to be characterised by elevated levels of specific metabolites, including Alternariol, dTMP, Oxymatrine, Gedunin, PC 36:4. Conversely, reductions in other metabolites, such as LPC O-24:0, PC 18:2_18:3, PC 19:2_19:2, Cholecalciferol and T-2 Triol, were also observed. Correlation analyses demonstrated that alveolar lavage microorganisms were closely associated with differential metabolites. Specifically, reductions in Cholecalciferol were associated with a variety of high-abundance flora and involved in vitamin digestion and absorption pathways. Furthermore, reductions in cholecalciferol may serve as a robust predictor of early-stage lung cancer. These findings provide new predictive biomarkers for early-stage lung cancer manifested by SPN, which is clinically important and requires further study of the potential mechanisms of action and function of the targets.

## 1 Introduction

Lung cancer is the most lethal malignant tumor globally, with the majority of patients diagnosed in the middle to late stages of the disease (Siegel et al., 2020; Sung et al., 2021). Non-small cell lung cancer (NSCLC), which represents the most prevalent form of lung cancer (85%), is associated with a prognosis that is strongly correlated with stage. Patients diagnosed with stage IA have a significantly higher five-year survival rate (70%) compared to those with stage IV (13%) (Aberle et al., 2011; Goldstraw et al., 2016), therefore the identification of early lung cancer is particularly important. In the early-stage of lung cancer, the disease typically manifests as a non-calcified solitary pulmonary nodule (SPN) (Rampinelli et al., 2016), which is defined as a round, nontransparent lesion isolated within the lung, has a maximum diameter of 3 cm, and is surrounded and completely encapsulated by lung parenchyma (Harzheim et al., 2015). The increased utilisation of low-dose CT has led to a rise in the detection of SPNs (Zhang et al., 2023), although the majority of these are ultimately identified as benign conditions, including infections, inflammations and vascular lesions. However, a proportion of SPNs are subsequently confirmed to be lung cancer (Larici et al., 2017). The low-dose CT scan is not sufficiently specific for use in screening for malignant SPNs (Boiselle, 2013; Church et al., 2013). It is therefore evident that the identification of early-stage lung cancer with SPN as the manifestation through the search for biomarkers with high sensitivity and specificity provides an invaluable reference point for medical decision-making.

Multi-omics combination studies are currently a prevalent methodology for investigating a spectrum of diseases, including genomics, proteomics, microbiomics and metabolomics (Song et al., 2019; Krug et al., 2020). The objective of metabolomic research is to identify differential metabolites within the body, which are then used as biological markers for disease (Bamji-Stocke et al., 2018). Studies have been conducted on the early detection, subtype differentiation, and mechanistic investigations of diseases using liquid chromatography (LC). Metabolites including hypoxanthine, L-tryptophan and indoleacetic acid have been identified as potential biomarkers for NSCLC (Ruiying et al., 2020). However, metabolites are susceptible to alteration by factors such as diet and nutritional status (Zhang et al., 2018). The involvement of microorganisms in the occurrence and development of NSCLC may be attributable to their effects on processes such as inflammation and metabolism (Wong-Rolle et al., 2021). A distinct intestinal microbiome has been identified in early-stage lung cancer, with microbial composition linked to tumour stage and subtype (Zheng et al., 2020). The difficulty of accurately elucidating comprehensive changes in organisms using a single omics analysis has prompted a gradual increase in comprehensive analyses of microbiomics and metabolomics. At present, multi-omics comprehensive studies on lung cancer are primarily concentrated on the analysis of intestinal microorganisms and serum metabolomics (Vernocchi et al., 2020; Ni et al., 2021; Ni et al., 2023).

Given the lungs are the host’s primary site of gas exchange, they are regarded as possessing a distinct microbiome when compared to the gut. This is evidenced by the lower number of colonies observed in the lungs (Mathieu et al., 2018). Local microecological studies of the lungs have indicated a strong correlation between microecological imbalance and the development of lung cancer. The characteristics of the local microbial communities in the lungs of lung cancer patients are as follows: the total number of these communities is greater, but the alpha diversity and bacterial abundance are reduced (Gomes et al., 2019). There is a paucity of research examining the local microbiome and metabolomics of lung cancer patients with SPN as the primary manifestation. In this study, a lobectomy specimen was employed for the purpose to perform bronchoalveolar lavage of the subsegment in which the SPN was located. The alveolar lavage fluid was subjected to 16S rDNA amplicon sequencing and metabolomic research with the objective of exploring the differences in the local lung microbiome and metabolites between lung cancer patients with SPN manifestations and those with benign SPN.

## 2 Materials and methods

### 2.1 Study participants

The study included 52 participants from the Department of Cardiothoracic Surgery, Mianyang Central Hospital, Sichuan Province. The imaging characteristics of all patients met the diagnostic criteria for SPN as outlined in the 2017 Fleischner Society Guidelines for the Management of Lung Nodules (MacMahon et al., 2017). All patients were diagnosed for the first time and underwent surgical resection to clarify the pathology type. The group included 31 patients with early-stage non-small-cell lung cancer (malignant), and 21 benign lung nodules (benign). There was no statistically significant difference in baseline data between the groups(P>0.05). Following the provision of written informed consent by all patients prior to surgery, alveolar lavage fluid was collected in accordance with a protocol approved by the ethics committee, and data pertaining to the patients’ clinical parameters was collated. Exclusion criteria included (1) the administration of antibiotics within the previous three months, (2) the presence of chronic respiratory infections and metabolic disorders, and (3) a history of definite pulmonary infections within the previous three months.

### 2.2 Sample collection and storage

Following the completion of surgical procedures, all subjects underwent lavage of the subsegment in which the SPN was situated in the excised lung tissue within a sterile operating theatre under the same quality control parameters. The alveolar lavage fluid was subsequently collected, and the duration of the operation was monitored to ensure it fell within the two-minute threshold post-lobectomy. The gathered specimens were then transferred to a refrigerated (-80°C) storage facility (Wells et al., 2019).

### 2.3 BALF microbiological analysis (16S rDNA sequencing)

#### 2.3.1 DNA extraction

The total deoxyribonucleic acid (DNA) from the disparate samples was extracted via the CTBA methodology, after which a 1% agarose gel electrophoresis was employed for the analysis of the purity and concentration of the DNA.

#### 2.3.2 Extracting and purifying the PCR product

Corresponding regions were amplified with the following primers: 515F and 806R (16SV4), 528F and 706R (18SV4), ITS5-1737F and ITS2-2043R (ITS1), and all PCR mixes were supplemented with Phusion^®^ PCR Master Mix (New England Biolabs), primers and genomic DNA templates, initial denaturation at 98°C, 30 cycles at different temperatures and finally 72°C for 5 minutes.

#### 2.3.3 Library preparation and sequencing

The libraries were quantified by Qubit and Q-PCR. The libraries were qualified for PE250 machine sequencing using NovaSeq 6000.

#### 2.3.4 Microbiology data analysis

The final Amplicon Sequence Variants (ASVs) characterisation table was obtained by splitting the raw data, double-ended splicing, quality control and chimera removal, and noise reduction using the DADA2 module in QIIME2 software (version QIIME2-202202). Species annotation was performed using QIIME2 software, 16S and 18S from Silva 138.1 database and ITS from Unite v9.0 database, based on which clustering and grouping of sequences was performed. The distribution of relative abundance in Perl was plotted as a histogram using the SVG function according to the 10 most abundant species at different taxonomic levels (phylum, order, order, family, genus and species) for each sample. Venn diagrams were generated in R using the Venn diagram function to visualise common and unique information between different samples or groups. (Alpha, α) Diversity reflects the diversity and richness of communities using Shannon, Chaol index, R to plot species cumulative box plots and rank gradient curves. To analyse the complexity of community composition between experimental and control groups, (Beta, β) diversity analysis was performed in QIME2 based on weighted and unweighted distances, and Principal Component Analysis (PCA) and Principal Coordinate Analysis (PCoA) were performed using R software (V4.0.3). Histograms of the distribution of LDA values, evolutionary branching diagrams and comparisons of biomarkers between groups were obtained by LEfSe (LDA Effect Size) statistical analyses, which were used to discover and interpret high-latitude biomarkers. For random forest analysis based on species abundance, different numbers of species were selected by gradient for different taxonomic levels, random forest models were constructed and ROC curves were plotted. Each model was then cross-validated (10-fold by default) and important species were filtered by MeanDecreaseAccuracy and MeanDecreaseGin.

### 2.4 Metabolomic Analysis of BALF

#### 2.4.1 Testing of the metabolite samples

Each lavage sample was placed in an EP tube with 400μL of an 80% methanol aqueous solution, subjected to several centrifugations, and the resulting supernatant was collected for subsequent LC-MS analysis. QC samples were derived from an equal-volume mix of individual experimental samples, while blank samples were prepared with a 53% methanol aqueous solution in place of the experimental samples. The UHPLC-MS spectra were analysed using a Q Exactive™ HF/Q Exactive™ HF-X mass spectrometer (Thermo Fisher, Germany) and a Vanquish UHPLC chromatograph (Thermo Fisher, Germany). Samples were injected onto a Hypersil Goldcolumn (100×2.1 mm, 1.9μm) using a 12-min linear gradient at a flow rate of 0.2 mL/min. The eluents for the positive and negative polarity modes were eluent A (0.1% FA in Water) and eluent B (Methanol). The solvent gradient was set as follows: 2% B, 1.5 min; 2-85% B, 3 min; 85-100% B, 10 min;100-2% B, 10.1 min;2% B, 12 min. Q ExactiveTM HF mass spectrometer was operated in positive/negative polarity mode with spray voltage of 3.5 kV, capillary temperature of 320°C, sheath gas flow rate of 35 psi and aux gas flow rate of 10 L/min, S-lens RF level of 60, Aux gas heater temperature of 350°C.

#### 2.4.2 Data processing and metabolite identification

The downstream data files (i.e.raw files) were imported into CD3.3 library search software for processing. Each metabolite was briefly screened for retention time, mass-to-charge ratio, and other parameters. Thereafter, the peak area was corrected with the first quality control (QC) sample to ensure more accurate identification. Finally, the peaks were extracted by setting the information. The parameters for this were 5 ppm of mass deviation, 30% deviation of signal intensity, minimum signal intensity, and the addition of ions. The data were then quantified by integrating the target ions, and the molecular formula was predicted and compared with the mzCloud (), mzVault, and Masslist databases by molecule peaks and fragmented ions. The quantification of target ions was followed by integration and molecular formula prediction by molecular ion peaks and fragmented ions. This was then compared with mzCloud (https://www.mzcloud.org/), mzVault and Masslist databases. Blank samples were used to remove background ions. The raw quantitative results were based on the formula: the original quantitative values of the samples/(The original quantitative results were then normalised according to the formula: original sample quantitative value/(sum of sample metabolite quantitative values/sum of metabolite quantitative values of QC1 samples) to obtain the relative peak area; compounds with a coefficient of variation of relative peak area greater than 30% in QC samples were deleted, and then the metabolites were identified and quantified relative to each other. (MS1 accurate quality vs. database values (ppm error), MS2 scoring values of spectra vs. matches in the mzCloud and mzvault databases, as detailed in **Supplementary Information**).

#### 2.4.3 Metabolomic data analysis

The identified metabolites were annotated using the Human Metabolome Database (HMDB), the Kyoto Encyclopedia of Genes and Genomes (KEGG), and the LIPID MAPS Structure Database (LIPIDMaps). The multivariate analysis employed the metaX tool to transform the data and subjected it to principal component analysis (PCA) and partial least squares discriminant analysis (PLS-DA). The resulting VIP values for each metabolite were then generated. Univariate sectioning was conducted using t-tests to ascertain statistical disparities (P-values) in metabolites between groups. The multiplicity of differences in metabolites between groups was expressed by (FoldChange, FC), with a default criterion of VIP > 1, P-value < 0.05 and FC ≥ 2 or FC ≤ 0.5. The R package ggplot2 was employed for the screening of target metabolites, with volcano and matchstick plots created using the combined metabolite parameters of VIP value, log2 (Fold Change) and -log10 (P-value). The clustering heatmap was plotted in the R language Pheatmap, while the bubble map was generated using ggplot2 in R. The metabolite functions and pathways were analysed using the KEGG database, and a metabolic pathway was considered to be enriched when x/n>y/n, and significantly enriched when the P-value of the metabolic pathway was <0.05.

### 2.5 Microbiome and metabolomics correlation analysis

Significantly disparate genera at the genus level, as determined by 16S rDNA, and markedly distinct metabolites, as identified by metabolomics analysis, were correlated using Pearson correlation coefficients. Heat maps were constructed to quantify the extent of the association between species diversity and metabolites in environmental samples.

## 3 Result

### 3.1 Clinical and demographic characteristics of the study participants

To study the microbiological and metabolite changes in alveolar lavage fluid of patients with early-stage lung cancer presenting as SPN, a group of 31 patients with early-stage lung cancer (malignant) and a group of 21 patients with benign lung nodules (benign) were enrolled. None of these patients had chronic lung disease, had no lung infections and had received antibiotic therapy in the 3 months prior to enrolment. There was no statistically significant difference in the baseline information of age, gender, body mass index (BMI) and smoking history between the two groups (P > 0.05). Group malignant was all metastasis-free early-stage NSCLC with the main pathological type of adenocarcinoma (29/31) and the control group was the group of benign lung nodules with the main pathological type of atypical adenomatous hyperplasia (n = 9). According to the imaging appearance of the nodules, they were classified as solid (13/52), subsolid (14/52) and ground-glass (25/52), and the characteristics of the subjects are shown in **Table 1**.

**Table 1:** General clinical comparison of group malignant and group benign.

Characteristics	Malignant nodule (n = 31)	Benign nodule (n = 21)	P value
Age (mean ± SD)	58.23± 10.59	55.10 ± 14.26	0.368
Male/female (No.)	15/16	7/14	0.281
BMI (kg/m2) (mean ± SD)	24.65 ± 2.18	23.56 ± 4.32	0.236
Tumor type, n (%)			
ADC	29	–	–
SCC	2	–	–
Nodule type			0.569
ground-glass nodule	14	11	
subsolid nodule	10	4	
solid nodule	7	6	
Smoking status, n (%)			0.292
Smoker	10	4	
Non-smoker	21	17	
Tumor metastasis, n (%)			
Non-metastasis	31	–	–
Metastasis	0	–	–
Family history, n (%)	0	0	–

### 3.2 Microbiological analysis of BALF

#### 3.2.1 The distribution of microorganisms in early lung cancer and benign lung nodules

**Figure 1:**
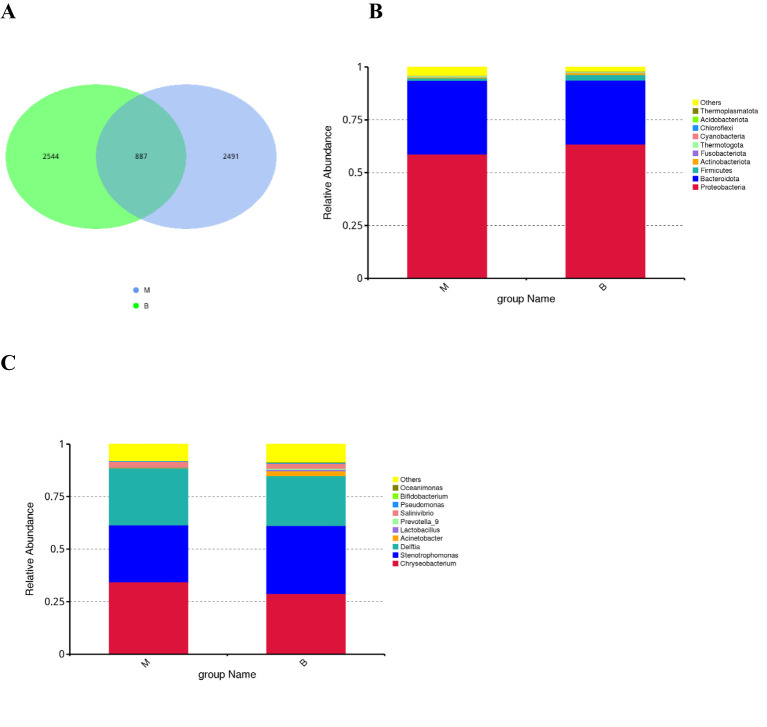
Early-stage lung cancer and benign lung nodules have different microbial distributions **(A)** The Venn diagram shows unique and common ASVs in group benign and group malignant. The top 10 representative species and their proportions in the two groups at the level of phylum **(B)**, and genus **(C)**.

The number of common and unique ASVs between group malignant and group benign was visualised by plotting Veen diagrams by ASV (**Figure 1A**). A total of 5922 ASVs were identified, with 887 ASVs common to both groups, of which 2,491 were unique to the malignant group and 2,544 to the benign group. At the phylum level, the main flora composition of BALF in the two groups was *Proteobacteria*, *Bacteroidetes* and *Firmicutes*, with being more abundant in group malignant (relative abundance 34.83%) than in group benign (relative abundance 30.24%), and *Firmicutes* being enriched in benign nodules. *Fusobacteriota* was also increased in group malignant compared with the group benign (**Figure 1B**). At the genus level, the group benign still had a richer microbial composition. *Chryseobacterium*, *Stenotrophomonas* and *Delftia* were the predominant lung microorganisms shared by the two groups, and *Chryseobacterium* and *Delftia* were significantly more abundant in the group malignant, *Acinetobacter*, *Prevotella_9*, *Bifidobacterium* and *Lactobacillus* were enriched in the group benign (**Figure 1C**).

#### 3.2.2 Alpha diversity and beta diversity of alveolar lavage flora

**Figure 2:**
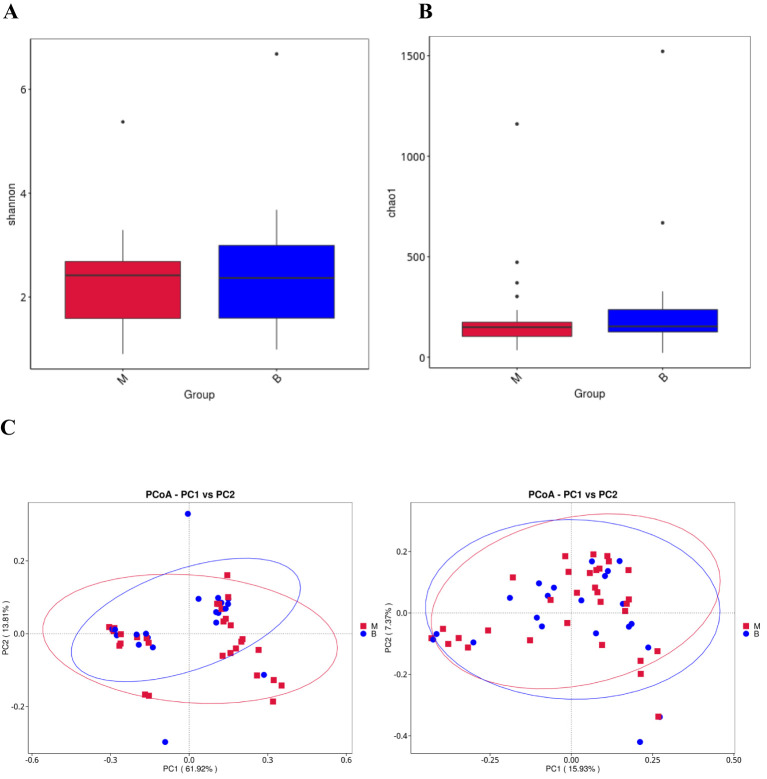
Alveolar lavage flora alpha diversity and beta diversity **(A)** Shannon diversity index of group benign and group malignant. **(B)** Chao 1 index of group benign and group malignant. **(C)** Beta diversity of the genera analysed by weighted UniFrac PCoA and unweighted UniFrac PCoA.

α-diversity reflects the richness and evenness of microbial communities within a sample, while β-diversity quantifies the diversity of microbial community composition between groups (Walters and Martiny, 2020). The Shannon index, which describes community richness, showed no significant difference between the two groups (group M vs. group benign, P = 0.439, **Figure 2A**). In addition, the Chao 1 index (group malignant vs. benign, P = 0.441, **Figure 2B**) was also not statistically different between the two groups. We then evaluated the β diversity of the two groups by PCoA analysis based on the weighted unifrac distance and unweighted unifrac distance, and found that there were some differences between the two groups of lavage species, but they were not statistically significant (**Figure 2C**). Using alpha and beta diversity analyses, we found that the richness and diversity of the microbial composition of alveolar lavage fluid was similar between patients with lung cancer and patients with benign lung nodules.

#### 3.2.3 Diagnostic biomarkers for lung cancer in alveolar lavage fluid

**Figure 3:**
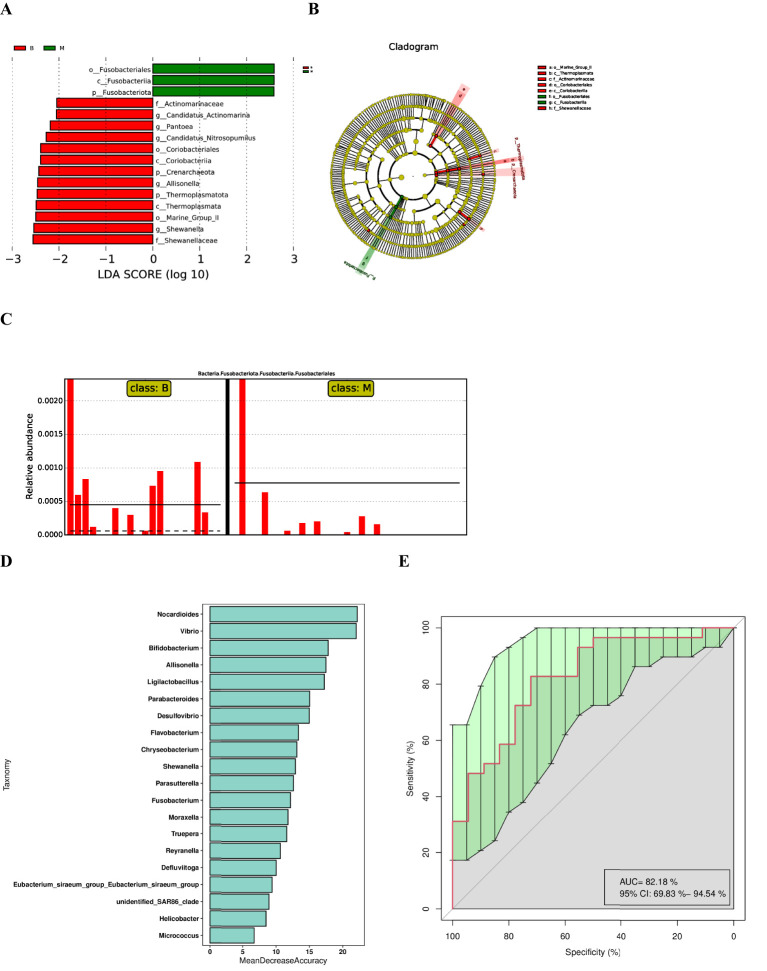
LEfSe analysis and diagnostic biomarker analysis by a random forest model. **(A)** Histogram of LDA value distribution(The magnitude of the effect of different species is represented by the LDA score, which is the length of the bars in the figure). **(B)** Cladogram. **(C)** Comparison of the abundances of significantly biomarker in group benign and group malignant. **(D)** Random forest model of the twenty microbial genera model. **(E)** ROC curve of the twenty microbial genera model.

The LDA Effect Size (LEfSe) was utilised to screen the classification units exhibiting significant variations between groups via the Kruskal-Wallis test. This procedure facilitated the identification of biomarkers with substantial differences between groups and the calculation of the discriminant weight of each classification unit exhibiting significant variations. the linear discriminant analysis (LDA) model.The LDA value reflects the contribution of a specific classification unit to the differences between groups, and the larger the value, the more important the classification unit is in distinguishing different groups. The LDA value reflects the degree of contribution of a particular classification unit to the differences between groups, and the larger the value, the more important the classification unit is in distinguishing different groups. Typically, LDA values greater than 2 are employed as the default threshold for identifying significant differences, and taxonomic units with high LDA values may serve as potential biomarkers. LDA Effect Size (LEfSe) analysis was performed to compare biomarker abundance between lung cancer and benign nodule groups, and the following three statistical conclusions were obtained: histograms of the distribution of LDA scores, evolutionary branching plots, and comparisons of biomarker abundance between groups. The histogram (**Figure 3A**) showed that there were 16 differential taxa at different classification levels, of which 3 were from group malignant and 13 from group benign, with log10 (LDA score) > 2. *Fusobacteria* had the highest LDA scores in the group malignant. Branching plots (**Figure 3B**) showed an increased abundance of *Fusobacteriales* from *Fusobacteria* in group malignant compared to group benign. In contrast, *Marine_Group_II*, *Enterobacterales* from the eye level were significantly enriched in the group benign. The relative abundance of biomarkers (**Figure 3C**) suggested that *Fusobacteriales* were significantly enriched in group malignant compared to group benign.

Further random forest analysis based on species abundance was performed to construct a model to discriminate lung cancer patients from benign lung nodules, which included 20 microbial genera (**Figure 3D**). The overall AUROC of the model was 82.18% (95%CI: 69.83%-94.54%) (**Figure 3E**), and the discriminatory power of genus-level *Nocardioides*, *Vibrio* and *Bifidobacterium* was significantly higher than that of other genera, suggesting that they are good diagnostic markers for lung cancer. Meanwhile, we found that the diagnostic value of *Fusobacterium* was also high, in line with the LEfSe results.

### 3.3 Metabolite differences between lung cancer group and benign lung nodule group

**Figure 4:**
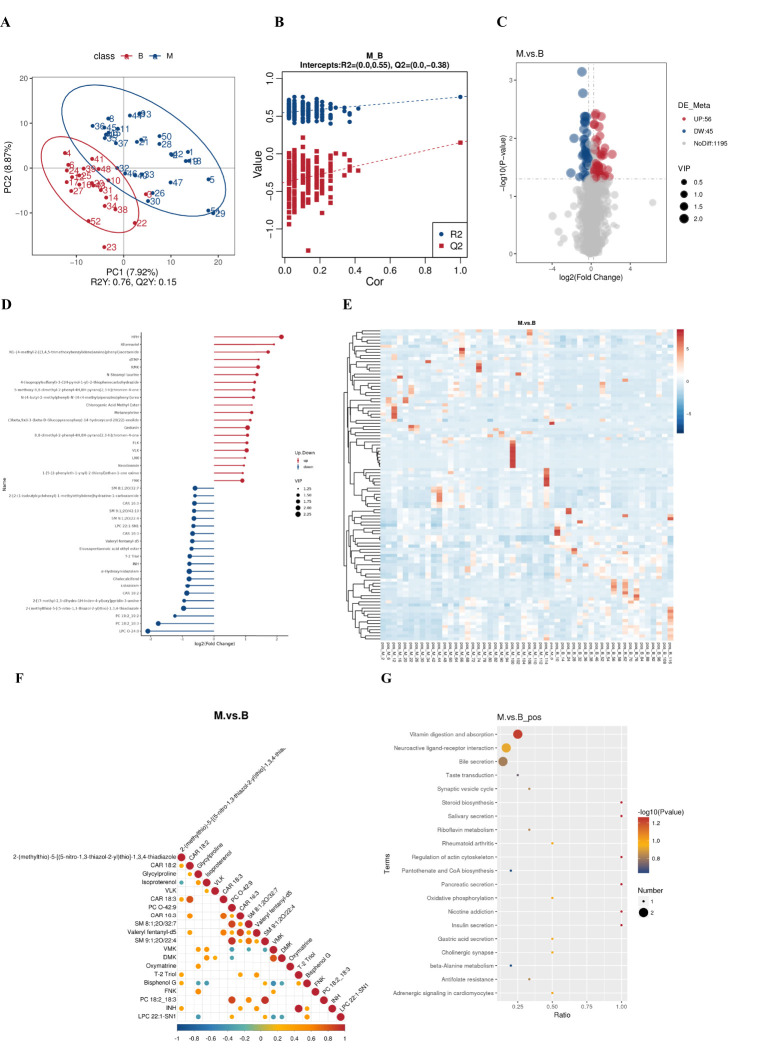
Metabolite differences between the groups. **(A)** PLS-DA score plot shows the difference in metabolites between groups. **(B)** Comparison of real and permuted model parameters in validation tests. **(C)** The significant different metabolites between group benign and group malignant by Volcano plot. **(D)** Matchstick diagram of differential metabolites. **(E)** Heat map of the differential metabolites in group benign and group malignant. **(F)** Correlation analysis of differential metabolites. **(G)** The KEGG pathway enrichment scatter plot displays important discriminatory metabolic processes of group benign and group malignant.(The color of the dots represents the P-value of the hypergeometric test; the smaller the value, the more reliable and statistically significant the test. The size of the dots represents the number of differential metabolites in the corresponding pathway; the larger the value, the more differential metabolites there are in that pathway).

Untargeted metabolomic analysis of BALF by LC-MS/MS was performed in order to determine whether there are differences in the metabolites of the local lung between patients diagnosed with lung cancer and those diagnosed with benign pulmonary lesions. A total of 1,917 metabolites were identified, comprising 1,296 positive ions and 621 negative ions. Among the cations, 101 differential metabolites were identified, of which 56 exhibited a significant upregulation and 45 demonstrated a significant downregulation. Partial Least Squares Discrimination Analysis (PLS-DA) was employed for the statistical selection of differential metabolites between groups. The results indicate the presence of differential metabolites between groups benign and malignant (**Figure 4A**). The ranking validation data does not exhibit the phenomenon of overfitting, and the R2Y and Q2 values are 0.57 and -0.38, respectively, confirming that the model has been effectively validated (**Figure 4B**). The differential metabolites were identified based on the Variable Importance in the Projection (VIP) of the PLS-DA model, with VIP > 1.0 and FC > 1.2 serving as the screening criteria. The results demonstrated the identification of a total of 101 ESI+ differential metabolites between the lung cancer group and the benign lung nodule group. The full list of differential metabolites is provided in **Figures 4C, D**. The principal upregulated differential metabolites were Alternariol, dTMP, Oxymatrine, Gedunin, PC 36:4, and so forth; the principal downregulated metabolites were LPC O-24:0, PC 18:2_18:3, PC 19:2_19:2, Cholecalciferol, T-2 Triol. Clustering analysis provides a more intuitive representation of the differences between individual samples (**Figure 4E**). The objective of the correlation analysis of differential metabolites is to ascertain the consistency of the trend of change in metabolites in relation to other metabolites. The correlation between each metabolite was analysed by calculating the Pearson correlation coefficient between all metabolites (**Figure 4F**). Subsequently, a KEEG pathway enrichment analysis (**Figure 4G**) revealed that the primary metabolic pathways involved in the differential metabolites between the two groups include: vitamin digestion and absorption, and neuroactive ligand-receptor interaction, among others.

### 3.4 Cross-correlation analysis between the microbiota and metabolites

**Figure 5:**
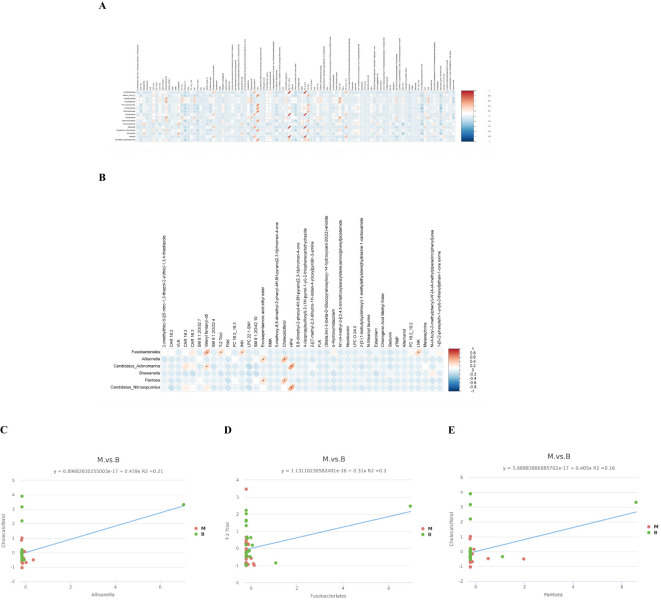
Cross-correlation analysis between the microbiota and metabolites. **(A)** A correlation heatmap of 16 ASVsand 101 differentially abundant metabolites at different levels is presented herewith. **(B)** A correlation heatmap of the top 20 metabolites with significant differences and the microorganisms *Allisonella*, *Candidatus*_*Actinomarina*, *Shewanella*, *Pantoea*, *Candidatus*_*Nitrosopumilus* and *Fusobacteriales*. (*p<0.05 in **A**, **B**). **(C)** The abundance of *Allisonella* was associated with low levels of Cholecalciferol. **(D)** The abundance of *Fusobacteriales* correlates with low levels of T-2 Triol. **(E)** The abundance of *Pantoea* was associated with low levels of Cholecalciferol. The horizontal coordinate represents the differentially abundant bacteria at the level of the 16S gene, and the vertical coordinate represents the differentially abundant metabolites. Red indicates a positive correlation, blue indicates a negative correlation, and an asterisk indicates statistical significance (P< 0.05).

In order to explore the functional relationship of the altered BALF microbiota and differentially accumulated BALF metabolites, we performed correlation analysis based on Pearson’s correlation coefficients. The top 16 ASVs with statistical difference annotated at the different level, and the above 101 differentially accumulated metabolites were included for analysis. It showed the metabolites were correlated with the microbiota of group malignant. The heatmap of the correlation is shown in **Figures 5A**. Besides, the results of the above microbiota study showed that *Allisonella*, *Candidatus*_*Actinomarina*, *Shewanella*, *Pantoea*, *Candidatus*_*Nitrosopumilus*.

in genus level and *Fusobacteriales* in order level were related to group malignant in **Figures 5B**. We also made the cross-correlation analysis between the three microbiotas with the differentially accumulated metabolites (**Figures 5C–E**).

## 4 Discussion

The prognosis of lung cancer patients is closely related to the timing of tumour diagnosis, and early diagnosis and treatment of lung cancer is key to improving prognosis (Aberle et al., 2011; Goldstraw et al., 2016). Low-dose CT cannot accurately identify malignant SPNs, so it is particularly important to find biomarkers for the early detection of malignant SPNs (Boiselle, 2013; Church et al., 2013). This study focused on analysing the local microbial and metabolic characteristics of patients with early-stage lung cancer presenting as SPNs and searching for potential biomarkers using a combined omics approach. The results are as follows: 1. At the phylum and genus levels, patients with early-stage lung cancer had fewer diverse microbial compositions than that in the benign nodule group, and has a unique microbial composition that may be used as a biomarker for the identification of early-stage lung cancer; 2. There are differences in metabolites between early-stage lung cancer patients and patients with benign lung nodules; 3. There is a certain correlation between local tumour microbes and metabolites in early-stage lung cancer patients, and microbes may be involved in the development of early-stage lung cancer through differential metabolites.

In our study, we found significant differences in the microbial composition of the two groups. At the phylum level, *Proteobacteria*, *Bacteroidetes* and *Firmicutes* were the main members of the lung microbes common to both groups of patients. Similar to our study, these microbes were found to be the main microbial components of healthy lungs (Man et al., 2017). The difference is that, We found that the abundance of *Bacteroidetes* was increased in lung cancer patients, and similar results were found in a study of the gut flora of lung cancer patients (Zhang et al., 2018). *Bacteroidetes* are thought to affect the host’s systemic inflammatory response and immunity by reducing the concentration of circulating short-chain fatty acids, thereby causing an imbalance in the structure of the intestinal flora in cancer patients and participating in tumourigenesis and development (Verdam et al., 2013; Gao et al., 2015). At present, the gut-lung axis has been confirmed to exist (Ma et al., 2022), Therefore, we speculate that *Bacteroidetes* may be involved in tumourigenesis in the local microenvironment of lung tumours. The present study found that lung cancer and benign lung nodules had comparable *Proteobacterial* levels, which contradicts Greathouse’s experimental findings. They found large numbers of Proteobacteria in lung cancer tissues that were immediately examined and in the adjacent non-tumour tissues of cancer patients, while no enrichment of Proteobacteria was found in healthy controls (Greathouse et al., 2018).This raises the question of whether tumours influence changes in the local and surrounding non-tumour tissue microenvironment. At the genus level, the benign lung nodule group still had a richer microbial composition. One study has identified an increase in the presence of *Chryseobacterium* in lung cancer tissues (Kovaleva et al., 2022). However, the diagnostic potential of this genus has yet to be confirmed, and the abundance of *Chryseobacterium* did not significantly correlate with the histological type of tumour. Additionally, the LDA results indicated an increase in the presence of *Chryseobacterium* in homozygous mice following the inhalation of BALF from NSCLC patients (Zheng et al., 2020). It is hypothesised that an increase in the abundance of *Chryseobacterium* in recurrent lung samples following the resection of early-stage adenocarcinoma is positively correlated with an upregulation of IL-2, IL-3, and IL-17 in the host transcriptome (Tsay et al., 2024).

Previous studies have found that the α diversity of sputum and alveolar lavage fluid bacterial communities is reduced in patients with previously diagnosed lung cancer, and that changes in diversity are considered an important indicator of malignant transformation (Hosgood et al., 2014; Lee et al., 2016). The α and β diversity analyses in this study suggest that compared with patients with benign lung nodules, there is no significant difference in the diversity and richness of alveolar lavage fluid bacterial communities in patients with early-stage lung cancer, contrary to the results of Wen’s study (Zeng et al., 2022). The reason for this finding may be explained by the results of another study suggesting that airway microorganisms change as lung cancer progresses (Marshall et al., 2022), Wen’s study included patients with advanced-stage lung cancer, whereas our study included patients only with early-stage lung cancer. Lobectomy specimens from lung cancer patients also suggest that the richness and uniformity of lavage microorganisms from patients are not different from those from controls (Zheng et al., 2021), suggesting that differences in sampling methods may affect the interpretation of microbiome data (National Academies of Sciences E, Medicine et al., 2017).

A LEfSe and random forest analysis confirmed that the significant predictive biomarkers for early-stage lung cancer are *Nocardioides*, *Vibrio*, *Bifidobacterium* and *Fusobacterium* at the genus level. Of these, *Fusobacterium* has high diagnostic value. *Fusobacterium* is the most significant genus of *Fusobacteria*, predominantly colonising the human oral cavity and colon. *Fusobacterium* nucleatum, which is highly abundant, has been detected in a variety of cancerous tissues, including those of the colorectum, oesophagus, breast, and pancreas (Kostic et al., 2012; Mitsuhashi et al., 2015; Hieken et al., 2016; Yamamura et al., 2016). *In vitro* experiments have demonstrated that the FadA adhesin of *Fusobacterium* nucleatum can stimulate the proliferation of human colon cancer cell lines, including HCT116, DLD1, and SW480 (Rubinstein et al., 2019). Additionally, *Fusobacterium* nucleatum has been demonstrated to facilitate the proliferation and invasion of colon cancer cells by upregulating microRNA 21 (miR21) (Yang et al., 2017). It has been demonstrated that *Fusobacterium* nucleatum is implicated in the chemoresistance of colon cancer. The precise mechanism may be that *Fusobacterium* nucleatum activates the Toll-like receptor 4 and myeloid differentiation primary response 88 signalling pathway, downregulates the expression of microRNA 18a and microRNA 4802, and induces the transformation of apoptosis to chemoresistance (Ramos and Hemann, 2017).

It is hypothesised that *Fusobacterium* has high predictive value for early-stage lung cancer. It is hypothesised that microorganisms exert an influence on tumour development through three principal mechanisms: the regulation of oncogenes or oncogenic pathways; the promotion of mutagenesis and tumourigenesis; and the alteration of the host immune system, which in turn affects tumour progression (Garrett, 2015).It is possible that microorganisms in the host environment may be causative agents of tumour formation, but they may also be mere passengers in the development of tumours. Our data indicate two possible scenarios: that they may act as promoters or that they may act as passengers. In order to gain a deeper understanding of the relationship between microorganisms and early-stage lung cancer, we also studied the differential metabolites of lung cancer and benign nodules.

Metabolites are the products of biological metabolism and are intrinsic to the host organism, providing a direct indication of the physiological processes occurring within the body (Johnson et al., 2012). Metabolomics is a growing area of research in the field of oncology. A variety of differential metabolites were identified in lung cancer patients, which suggests that metabolic disorders may be involved in the pathogenesis of lung cancer. The PLS-DA analysis revealed significant differences in the composition between the two groups. A total of 101 differentially accumulated metabolites were identified in cations, with 56 being significantly upregulated and 45 being significantly downregulated. Compared with benign lung nodules, the differentially accumulated metabolites Alternariol, dTMP, Oxymatrine, Gedunin, PC 36:4, and others in lung cancer patients exhibited significant upregulation. Alternariol (AOH) is a mycotoxin produced by the Alternaria species of fungus. Given its oestrogen-like properties, it has been linked to an increased risk of oesophageal cancer and endocrine disorders (Saleh et al., 2024). Deoxythymidine monophosphate (dTMP) plays a role in the development of tumours through a number of mechanisms. Deoxythymidine kinase (DTYMK) catalyses the conversion of dTMP to deoxythymidine diphosphate (dTDP), which represents a pivotal step in pyrimidine metabolism. Elevated DTYMK expression in tumours such as liver cell carcinoma (LIHC) and lung adenocarcinoma (LUAD) is associated with a poor prognosis. Furthermore, DTYMK expression levels are linked to the presence of immune cells within various cancer types. This indicates that dTMP, which is regulated by DTYMK, plays a significant role in tumour progression and immune response regulation across diverse cancer types (Lan et al., 2022);In triple-negative breast cancer (TNBC), cytoplasmic cytidylyl-5’-phosphate hydrolase (CT) induces apoptosis by hydrolysing dTMP, thereby creating a nucleotide imbalance. This imbalance results in the disruption of the tricarboxylic acid cycle, which in turn causes metabolic stress and cell death. This approach suggests the potential for targeting dTMP metabolism as a means of inducing tumour cell death (Kim et al., 2022);Gedunin represents a novel microtubule inhibitor that circumvents the typical mechanism of drug resistance observed in cancer therapy. It has been demonstrated that this agent exerts pronounced cytotoxic effects on a range of cell lines, including those of lung cancer (Khalid et al., 2022). Gedunin has been demonstrated to disrupt the interaction between Hsp90 and key proteins, including Beclin-1 and Bcl-2, thereby inducing increased apoptosis in A549 lung cancer cells (Hasan et al., 2020). The concentration of PC 36:4 is elevated in bladder cancerous tissue, and may potentially serve as a biomarker for the disease (Ho et al., 2022). A significant down-regulation was observed in a number of compounds, including LPC O-24:0, PC 18:2_18:3, PC 19:2_19:2, cholecalciferol and T-2 triol, in lung cancer tissue. LPC O-24:0 is a lysophosphatidylcholine, particularly LPC [16:0] and LPC [18:2], which were observed to be diminished in tumour tissue relative to healthy tissue. This depletion, in conjunction with the accumulation of other glycerophospholipids, indicates that LPC may serve as a diagnostic marker for differentiating between tumour and non-tumour tissue (Schmidt et al., 2020). The application of proteomics strategies has revealed the significance of lipid biomarkers, including LPC, in enhancing the sensitivity and specificity of UBC diagnosis. The incorporation of LPC into biomarkers has the potential to enhance diagnostic accuracy (Tabaei et al., 2023). The aforementioned studies indicate that LPC, including LPC O-24:0, may serve as promising tumour markers. However, further research is necessary to elucidate their precise diagnostic value. The metabolite phosphatidylcholine (PC) is indispensable for the maintenance of membrane integrity and the promotion of lipid signalling pathways, which are of paramount importance for the growth of cancer cells. In hepatocellular carcinoma (HCC), exercise-induced alterations in phosphatidylcholine (PC) metabolism, particularly PC 18:1/18:1, have been demonstrated to remodel the tumour microenvironment and reduce inflammation (Zhang et al., 2024). These findings indicate that alterations in polycarbonate species, including PC 18:2_18:3/PC 19:2_19:2, can also influence tumour biology by modifying the tumour microenvironment.

The metabolism of Cholecalciferol represents a classic metabolic pathway. A reduction in vitamin D levels has been demonstrated to elevate the risk of a number of cancers, including breast cancer, colon cancer, prostate cancer, and blood cell cancer. 58 (Tabaei et al., 2023). Vitamin D has been demonstrated to exert an inhibitory effect on the development of lung cancer by downregulating the expression of the histidine-rich calcium-binding protein (Carlberg and Muñoz, 2022). *In vitro* experiments have demonstrated that the combination of cisplatin nanoparticles and vitamin D3 can markedly suppress inflammatory processes and diminish the expression of tumour markers in mice with early-stage lung cancer. It is therefore reasonable to posit that a reduction in cholecalciferol levels is an effective predictor of early-stage lung cancer (Carlberg and Muñoz, 2022).

Vitamin D has been demonstrated to exert an inhibitory effect on the development of lung cancer by downregulating the expression of the histidine-rich calcium-binding protein (Liu et al., 2021). *In vitro* experiments have demonstrated that the combination of cisplatin nanoparticles and vitamin D3 can markedly suppress inflammatory processes and diminish the expression of tumour markers in mice with early-stage lung cancer (Radwan et al., 2021). It is therefore reasonable to posit that a reduction in cholecalciferol levels is an effective predictor of early-stage lung cancer. T-2 triol, a derivative of the T-2 toxin, is a trichothecene mycotoxin produced by *Fusarium* sp*ecies*, which has been studied for its potential effects on tumor development. T-2 triol and other metabolites, including HT-2 toxin and neosolaniol, demonstrated a notable intracellular accumulation in HepG2 cells, which contributed to the cytotoxicity of T-2 toxin. This accumulation resulted in increased necrosis and apoptosis in these cells, indicating that cell viability and proliferation were disrupted (Taroncher et al., 2020). Furthermore, KEGG enrichment analysis indicated that the metabolites were predominantly involved in signalling pathways, including neuroactive ligand-receptor interaction and vitamin digestion and absorption. This finding is also consistent with the observed differences in the metabolites.

The present study revealed a correlation between the microbiome and metabolites in alveolar lavage fluid through correlation analysis. However, the lack of a clear causal relationship precludes any definitive conclusions. Notably, *Allisonella* and *Pantoea*, which were highly abundant in tumour tissue, were both correlated with the low level of the metabolite Cholecalciferol. A reduction in vitamin D levels has been demonstrated to elevate the risk of various cancers (Carlberg and Muñoz, 2022). The differentially expressed metabolites identified in this study were predominantly associated with the vitamin digestion and absorption signalling pathway. The *Fusobacteriales* biomarker, which has been identified as a potential predictor of tumour development, was also found to be positively correlated with the downregulated metabolite T-2 triol. These findings collectively suggest that during the early-stages of lung cancer development, microbial activity may influence the metabolism of cholecalciferol via the vitamin digestion and absorption signalling pathway, which is intricately linked to tumour formation. As indicated by the results of the above-mentioned metabolic analysis, the presence of plant or fungal metabolites was indicated. Consequently, a comprehensive review of the entire study was conducted. Firstly, we performed alveolar lavage in strict accordance with aseptic procedures; we also examined the results of serum G and GM tests in both groups of patients, and the results were negative; we did not observe any fungi or hyphae in either group. Secondly, our study subjects were not treated with any specific drugs prior to surgical treatment; however, in the metabolite study we identified plant or fungal metabolites. We propose that the presence of these metabolites may be due to the following reasons: 1) Alternariol and T-2 triol are common mycotoxins in soil and air, which can be directly inhaled through respiration and deposited in the lungs. According to epidemiological studies, these toxins are found in high concentrations in the air in humid climates or agricultural regions (Sichuan is a humid agricultural province) (Täubel et al., 2011). Oxymatrine, the main alkaloid of the plant Sophora flavescens, can be retained in the pulmonary circulation by inhalation of dust containing Sophora flavescens (e.g. during the processing of traditional herbal medicines) or by ingestion (Pierce et al., 2020). Residues in the pulmonary circulation may occur after inhalation or ingestion. 2)Metabolite cross-reactivity: Some bacterial secondary metabolites may show structural similarities to fungal toxins in mass spectrometry assays, potentially leading to cross-recognition (Wakefield et al., 2017; Keller, 2019).

It is important to note that our study is not without limitations. Firstly, the total sample size is relatively small, and the study population is mainly composed of adenocarcinoma cases, which limits the generalizability of our findings to other early-stage lung cancers with different pathological types. Secondly, it is essential to consider the impact of smoking, living environment and dietary habits on microbial and metabolic profiles. It is therefore necessary to acknowledge that our findings may not be applicable to populations with different dietary environments and geographic locations. Furthermore, the observed findings were not validated in animal tests, which would have enabled a more comprehensive evaluation of the functions of the observed differential microbes and metabolites.

## 5 Conclusion

In conclusion, the results of this study suggest that patients with early-stage lung cancer have unique lung microbiota and significant differential metabolites. Furthermore, the multi-omics study revealed that certain lung microbiota in lung cancer patients may be associated with reduced Cholecalciferol, T-2 Triol. It is possible that early-stage lung cancer affects the structure and abundance of lung microbiota, thereby interfering with the metabolic homeostasis of the host. Lung microbiota and metabolites may therefore represent an entry point for the prediction and even treatment of early-stage lung cancer. However, further animal experiments are required to verify the possible targets identified in this study, which will provide a stronger theoretical basis for the prediction of early NSCLC with SPN as the manifestation.

## Data Availability

The original contributions presented in the study are included in the article/**Supplementary Material**. Further inquiries can be directed to the corresponding author.
