# Supplementary material for: Integrative multi-omics analysis of the microbiome and metabolome in bronchoalveolar lavage fluid from patients with early-stage lung cancer
Source: Front Cell Infect Microbiol. 2025 Apr 28;15:1513270. doi: 10.3389/fcimb.2025.1513270 (PMC12066597; doi:10.3389/fcimb.2025.1513270)
Supplement: Supplementary file 1 [file Table1.docx]

**Supplementary Information**

| **Compound_ID** | **Name** | **Formula** | **PPM** | **Molecular Weight** | **RT [min]** | **m/z** | **mzCloud_Results** | **mzVault_Results** | **MassList_Results** | **mzCloud Best Match** | **mzVault Best Match** | **IDC level** |
| --- | --- | --- | --- | --- | --- | --- | --- | --- | --- | --- | --- | --- |
| Com_3923_pos | Alternariol | C14 H10 O5 | 11.0575 | 258.05 | 5.088 | 259.0573 | Invalid mass | No results | No results | 61.9 |  | 2 |
| Com_7299_pos | PC 19:2_19:2 | C46 H84 N O8 P | 4.552011 | 809.5898 | 10.635 | 810.5971 | No results | Full match | No results |  | 73.7 | 2 |
| Com_9498_pos | dTMP | C10 H15 N2 O8 P | 4.695044 | 322.0551 | 5.111 | 345.0443 | No results | No results | Full match |  |  | 3 |
| Com_15680_pos | Gedunin | C28 H34 O7 | 0.359637 | 482.2303 | 5.616 | 483.2376 | Full match | No results | No results | 69.6 |  | 2 |
| Com_18554_pos | Cholecalciferol | C27 H44 O | 0.09374 | 384.3392 | 6.756 | 385.3465 | Full match | No results | No results | 72.4 |  | 2 |
| Com_24752_pos | T-2 Triol | C20 H30 O7 | 5.110686 | 382.1972 | 8.943 | 383.2045 | Invalid mass | No results | No results | 56.9 |  | 2 |
| Com_31030_pos | Oxymatrine | C15 H24 N2 O2 | 0.915999 | 264.184 | 5.105 | 265.1914 | Full match | No results | No results | 42.5 |  | 2 |
| Com_46049_pos | PC 36:4 | C44 H80 N O8 P | 28129.16 | 803.5468 | 8.254 | 804.5541 | No results | Invalid mass | No results |  | 51.7 | 2 |
| Com_2627_pos | PC 18:2_18:3 | C44 H78 N O8 P | 23103.72 | 797.5569 | 10.745 | 798.5641 | No results | Invalid mass | No results |  | 65.5 | 2 |
| Com_10729_pos | LPC O-24:0 | C32 H68 N O6 P | 0.278908 | 593.4783 | 9.681 | 594.4855 | No results | Full match | No results |  | 96.3 | 2 |

**Table 1.** **Metabolite quantification results**

* Compound_ID: metabolite ID; Name: metabolite's descriptive information; Formula: metabolite's molecular formula; PPM: parts per million; Molecular Weight: molecular weight; RT [min]: retention time; m/z: mass-to-charge ratio; The identification of metabolites is based on the matching of detected m/z with m/z generated from metabolic databases. Our non-targets rely primarily on the mzCloud database paired with the mzVault database and the Masslist database. In instances where the mzCloud database identifies substances as either Full Matches, or Invalid Masses, this is indicated in the "mzCloud_Results" column. The substance has been identified as non-target metabolism according to the standards set out in the Metabolomics Standards Initiative (MSI) standard IDC level. Furthermore, mzcloud and mzvault have been identified at level 2, and masslist at level 3. (This section can be obtained by contacting the corresponding author by email if more data is required)

**A**


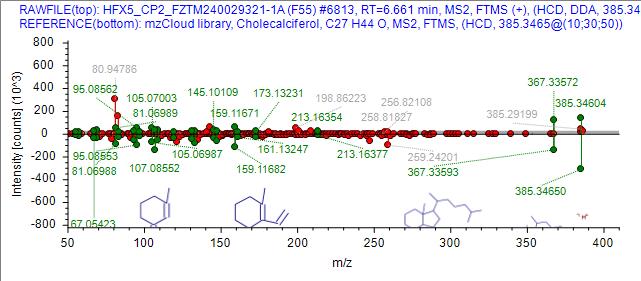


**B**


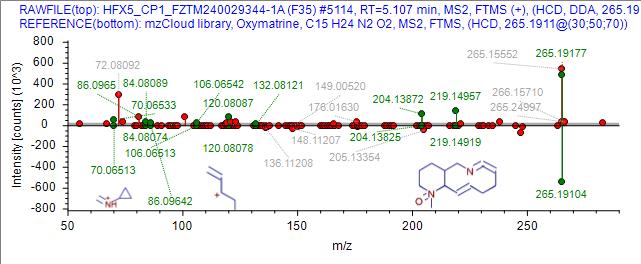


**Figure 1** The upper panel is indicative of the mirror image of metabolites Cholecalciferol and Oxymatrine, respectively.

**A B**


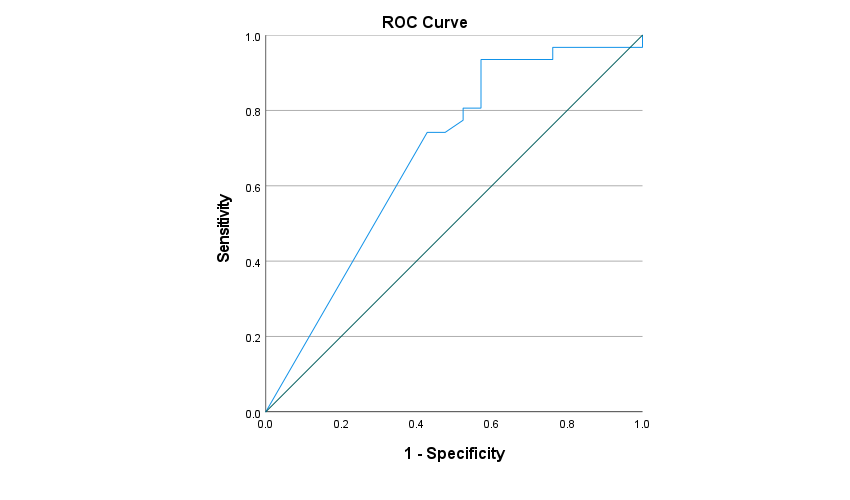

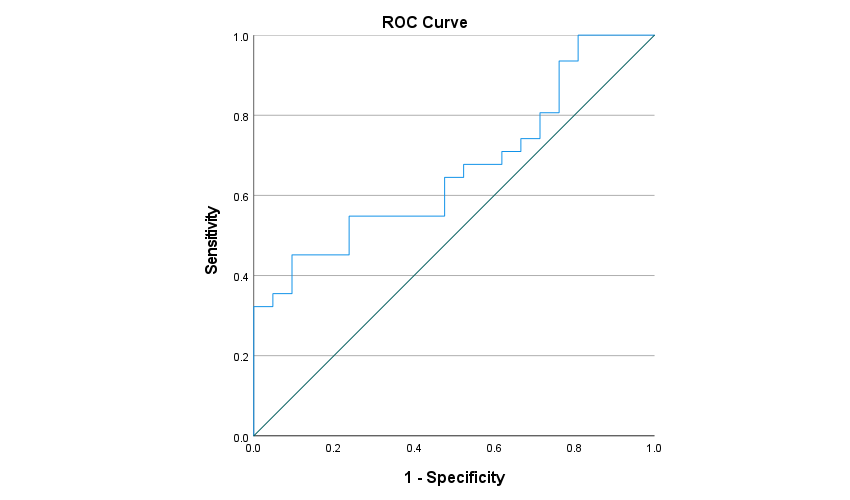


**C**


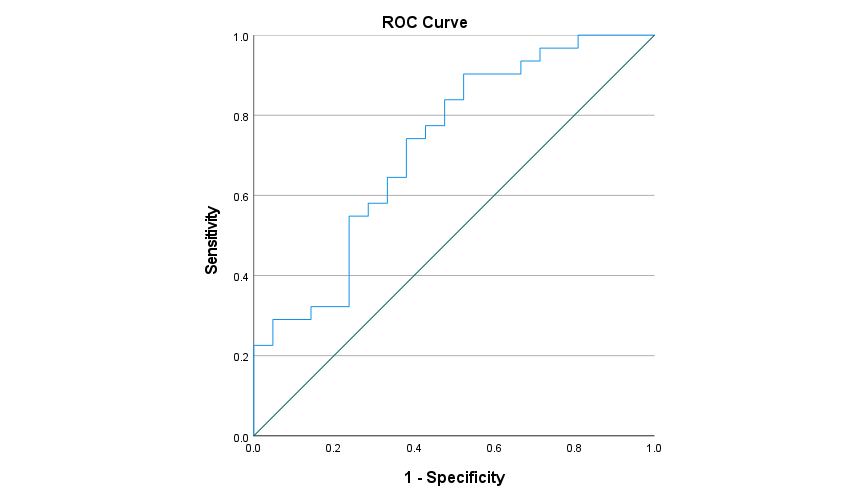


**Figure 2** The predictive value of *Fusobacteriales* and cholecalciferol for malignant SPN by using ROC curve. (A) The AUC of Fusobacteriales for discriminating malignant and benign lesions was 0.677 (95% CI: 0.523–0.832; p < 0.05). (B) The AUC of cholecalciferol for discriminating malignant and benign lesions was 0.665 (95% CI: 0.518–0.812; p < 0.05). (C) The combination of *Fusobacteriales* and cholecalciferol was used to differentiate malignant from benign. The area under the curve is 0.722 (95% CI: 0.579–0.865; p < 0.05)
